# New Insight into Therapies Targeting Angiogenesis in Hepatocellular Carcinoma

**DOI:** 10.3390/cancers11081086

**Published:** 2019-07-31

**Authors:** Monica Mossenta, Davide Busato, Lorena Baboci, Federica Di Cintio, Giuseppe Toffoli, Michele Dal Bo

**Affiliations:** 1 Experimental and Clinical Pharmacology Unit, Centro di Riferimento Oncologico di Aviano (CRO), Istituto di Ricovero e Cura a Carattere Scientifico (IRCCS), 33081 Aviano (PN), Italy; 2 Department of Life Sciences, University of Trieste, 34127 Trieste, Italy

**Keywords:** HCC, angiogenesis, small-molecule kinase inhibitors, immunotherapy

## Abstract

Hepatocellular carcinoma (HCC) is a malignancy characterized by neoangiogenesis that is determined by an augmented production of proangiogenesis factors by tumor and adjacent cells. This unbalanced angiogenesis process is a key feature of HCC carcinogenesis and progression. Proangiogenic factors also have a relevant role in the generation and maintenance of an immunosuppressive tumor microenvironment. Several therapeutic options for HCC treatment are based on the inhibition of angiogenesis, both in the early/intermediate stages of the disease and in the late stages of the disease. Conventional treatment options employing antiangiogenic approaches provide for the starving of tumors of their blood supply to avoid the refueling of oxygen and nutrients. An emerging alternative point of view is the normalization of vasculature leading to enhance tumor perfusion and oxygenation, potentially capable, when proposed in combination with other treatments, to improve delivery and efficacy of other therapies, including immunotherapy with checkpoint inhibitors. The introduction of novel biomarkers can be useful for the definition of the most appropriate dose and scheduling for these combination treatment approaches. The present review provides a wide description of the pharmaceutical compounds with an antiangiogenic effect proposed for HCC treatment and investigated in clinical trials, including antibodies and small-molecule kinase inhibitors.

## 1. Introduction

Hepatocellular carcinoma (HCC) is the sixth most common cancer worldwide. HCC usually develops from a pre-existent liver disease, commonly a cirrhotic state, and is related with well-defined risk factors including chronic viral types B and C hepatitis, alcohol abuse and aflatoxin exposure [1,2].

HCC is characterized by a high presence of vascular abnormalities with aberrant microvasculature generated by the arteriogenesis and capillarization [3,4]. Generally, the vasculature is less dense than that of normal liver and immature abnormal leaky tumor vessels can be found, giving rise to interstitial hypertension, edema and tumor hypoxia with necrotic regions [3,5,6,7]. In turn, in a vicious circle, hypoxia can re-stimulate angiogenesis and ultimately tumor growth [3,6,8,9].

Several therapeutic options for HCC treatment are based on the inhibition of angiogenesis, both in the early/intermediate stages of the disease and in the late stages of the disease [9]. Transarterial chemoembolization (TACE) is a locoregional treatment used in the early/intermediate stages [10]. Systemic therapies with small molecules acting as kinase inhibitors exert an antiangiogenic function by targeting various tyrosine kinases involved in neovascularization. These small molecules, in particular sorafenib, are frequently used in the late stages of the disease [9,11]. Moreover, the humanized anti-vascular endothelial growth factor (VEGF) antibody bevacizumab has been proposed in combination with conventional chemotherapy and radiotherapy [12,13].

Conventional treatment options employing antiangiogenic approaches provide for the starving of tumors of their blood supply to avoid the refueling of oxygen and nutrients [3]. In this context, tumors with excessively reduced blood vessels could reach a hypoxic state associated with an increased invasiveness and capability to metastasize [3]. An emerging alternative point of view is the normalization of vasculature leading to augmented tumor perfusion and oxygenation, potentially capable, when proposed in combination treatment approaches, to improve delivery and efficacy of other therapies, including immunotherapy with checkpoint inhibitors [3,6,14,15].

**Figure 1:**
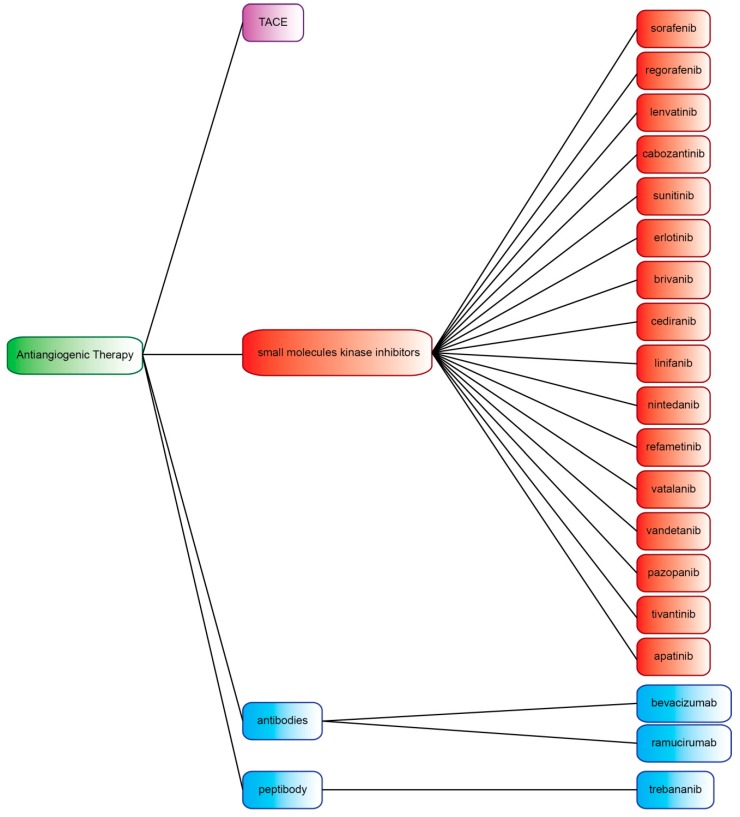
Antiangiogenic therapeutic strategies considered in the present review. TACE: transarterial chemoembolization.

**Figure 2:**
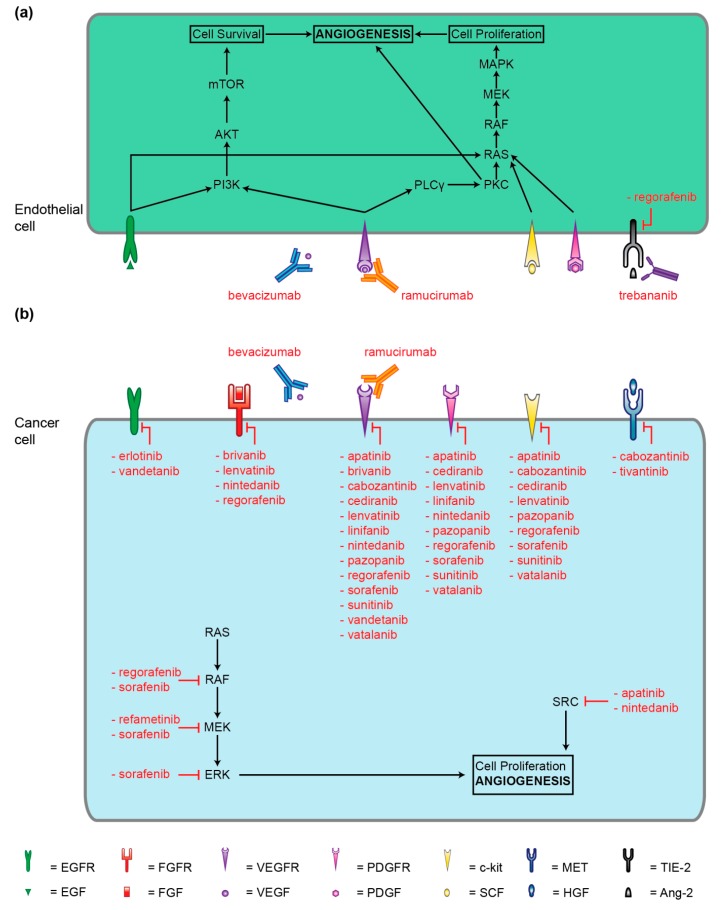
Molecular targets of antiangiogenic therapies. (**a**) molecur targets in endothelial cells, (**b**) molecular targets in tumor cells.

In the present review, we will describe the main molecular mechanisms of neoangiogenesis potentially influencing the interactions between tumor, tumor microenvironment and immune system cells. Moreover, we will give a wide description of the pharmaceutical compounds with an antiangiogenic effect proposed for HCC treatment and investigated in clinical trials (Figure 1 and Figure 2, Table 1).

## 2. Molecular Mechanisms of Neoangiogenesis Involved in Pathogenesis and Progression of HCC

HCC develops from a dysplastic nodule through the acquisition of an increasing number and density of arteries without the associated bile ducts [3,5,16,17]. This is a key step in hepatocarcinogenesis that is determined by an unbalanced angiogenesis process with an augmented production of proangiogenesis factors (drivers of vessel growth and maturation) by tumor cells and adjacent cells, including VEGF, platelet-derived growth factor (PDGF), placental growth factor, angiopoietins, hepatocyte growth factor, endoglin, transforming growth factor, basic fibroblast growth factor, and a diminished production of inhibitors such as angiostatin, endostatin, thrombospondin-1 [3,6,9]. Moreover, downstream intracellular signaling and endothelial cell tyrosine kinases are activated by factors able to induce proangiogenesis through both mitogen-activated protein kinase (MAPK) and extracellular-signal-regulated kinase (ERK) (MEK) and phosphatidylinositol-3-kinases (PI3K)/protein kinase B (Akt)/mammalian target of rapamycin (mTOR) pathways [9,18].

**Figure 3:**
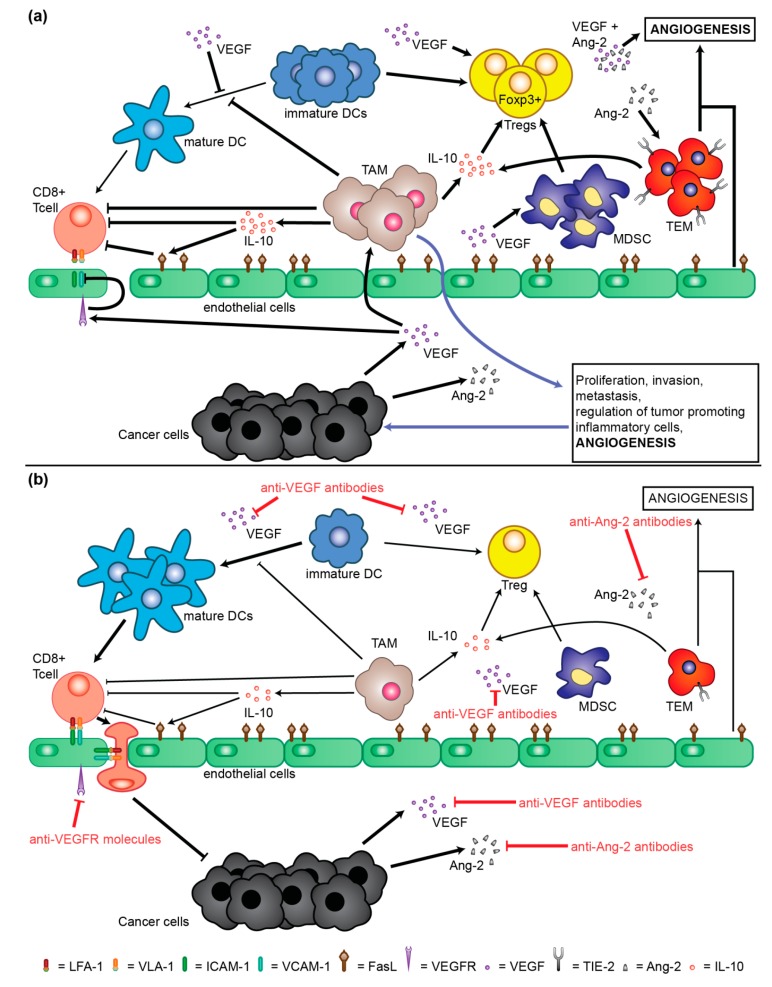
Immunosuppressive role of angiogenic factors. (**a**) Schematic representation of tumor microenvironment actors in a non-treated situation. (**b**) Schematic representation of tumor microenvironment actors in a treated situation. Abbreviations: Ang-2 = angiopoietin-2; DC = dendritic cell; FasL = Fas ligand; ICAM-1 = intercellular adhesion molecule-1; IL-10 = interleukin-10; LFA-1 = lymphocyte function-associated antigen 1; MDSC = myeloid-derived stem cells; TAM = tumor associated macrophages; TEM = TIE-2 expressing monocyte; TIE-2 = angiopoietin receptor; Tregs = regulatory T lymphocytes; VCAM-1 = vascular cell adhesion molecule-1; VEGF = vascular endothelial growth factor; VEGFR = vascular endothelial growth factor receptor; VLA-1 = very late antigen-1.

Various indications have arisen regarding the link between the immune system and angiogenesis in the tumor microenvironment context (Figure 3). Abnormal angiogenesis reduces the number and the functionality of tumor infiltrating lymphocytes (TIL), in particular for the presence of leaky nascent vessels and loose pericyte coverage resulting in a high interstitial fluid pressure, which determines a greater pressure difference to overcome for the infiltration of T cells. There is a well-recognized role of VEGF as key mediator in the evasion of the immune-surveillance by tumor cells in the context of an immunosuppressive tumor microenvironment [6,14,19]. T cell diapedesis occurs through the interaction between lymphocyte function-associated antigen 1 (LFA-1, expressed by T cells) and intercellular adhesion molecule 1 (ICAM-1, expressed by endothelial cells) and between very late antigen-1 (VLA-1, expressed by T cells) and vascular cell adhesion molecule 1 (VCAM-1, expressed by endothelial cells) [14]. VEGF is capable to generate a defective clustering of ICAM-1 and VCAM-1 at the endothelial surface, that is involved in the inhibition of T cell infiltration [14,20]. VEGF is also capable to induce proliferation of regulatory T lymphocytes (Tregs) [21]. Moreover, VEGF is associated with the accumulation of myeloid-derived suppressor cells (MDSC) which in turn can induce the development of forkhead box P3 (Foxp3+) Tregs [6,22]. High VEGF levels are also associated with a decrease of dendritic cells capable to reach a mature state. The presence of immature dendritic cells can contribute to the differentiation and proliferation of Tregs [6,14,19]. Tumor-associated macrophages (TAM) can also be induced by VEGF [6,23,24]. Another proangiogenic factor that has an immunosuppressive role is angiopoietin-2 (Ang-2). In particular, the expression of Ang-2 by tumor cells can induce the recruitment of TIE-2-expressing monocytes (TEM) which in turn are responsible for the release of IL-10 [25,26]. This IL-10 production is associated with the suppression of T cell proliferation, an increment in the CD4^+^/CD8^+^ T cell ratio, and the expansion of Foxp3+ Tregs [27]. In the mechanisms limiting the infiltration of T cells into the tumor, a relevant role is also carried out by Fas Ligand (FasL) as a mediator of T cell apoptosis. In fact, endothelial cells expressing FasL on tumor endothelial barrier can selectively kill effector T cells rather than Tregs [28]. Thus, the expression of FasL is associated with the absence of CD8^+^ T cells into the tumor, whereas Tregs are still present due to the high expression of cellular FLICE-inhibitory protein [28]. Tumor hypoxia upregulates known inhibitory molecules of the antitumor immune response such as programmed death-ligand 1 (PD-L1), IL-6, IL-10 and indoleamine 2, 3-dioxygenase (IDO) [29]. A hypoxic state of the tumor also induces the upregulation of C-C motif chemokine 11 (CCL11) and chemokine (C-C motif) ligand 28 (CCL28) capable to recruit Tregs into the tumor microenvironment [30]. Moreover, tumor hypoxia promotes the polarization of TAM to M2-like phenotype [31].

Antiangiogenic treatments can induce or increase the trafficking of T cells reactive versus specific tumor antigens or of other effector cells of the immune system [32]. In particular, the interaction between the endothelium and T cells can be reverted by the blockade of VEGF through the VCAM-1 and ICAM-1 role [32]. Furthermore, a reduced hypoxia preferentially induces polarization of TAM to M1-like phenotype [15]. Moreover, an improved perfusion downregulates PD1-PDL1 immune inhibitory signals induced by a hypoxic state [15]. The use of kinase inhibitors decreases PD1 expression in tumor infiltrating T cells [33], significantly increase the infiltration of CD8^+^ and CD4^+^ T cells in tumors in in-vivo mouse models [15,32,33]. In addition, the number of Tregs are reduced upon kinase inhibitor treatments as well as the number of MDSC and their suppressive function [22,33]. Moreover, anti-VEGF agents could inhibit the expression of immune checkpoint inhibitors (ICIs) such as PD-L1, cytotoxic T-lymphocyte-associated protein 4 (CTLA-4) and T-cell immunoglobulin and mucin-domain containing-3 (TIM-3) in infiltrating CD8^+^ T cells [34]. Finally, the use of antibodies blocking FasL can increase CD8^+^ T cell infiltration into the tumor [28]. On the other hand, an excessive inhibition of angiogenesis can generate hypoxia in the tumor microenvironment which, in turn, can contribute to an increase of immunosuppression [3,19,32,35].

## 3. Transarterial Chemoembolization (TACE)

TACE is characterized by the injection of lipidol emulsified with chemotherapeutic drug into the central vessels that feed the liver tumor nodules with the subsequent embolization of the same vessels to induce both cytotoxic and ischemia effects. TACE is used to treat patients with Barcelona Clinic Liver Cancer (BCLC) 0/A until liver transplant is available, and patients with BCLC stage B or C as non-curative treatment [10]. TACE can enhance the median survival from 16 to 19–20 months in patients without the possibility of surgical or ablation treatments and with intermediate stage HCC (BCLC stage B) [10]. On the other hand, reactivation of angiogenesis may occur, probably due to the hypoxemia induced by TACE, and neovascularization can occur in the periphery [10]. Combination therapies using both TACE and antiangiogenic agents may be useful to enhance the therapeutic effects (Table S1) [10].

## 4. Small-Molecule Kinase Inhibitors

In the last years, protein kinases have been considered one of the most interesting targets for numerous diseases, including cancer [36], and several kinase inhibitors have been developed [37]. In this context, Imatinib was the first kinase inhibitor approved by Food and Drug Administration (FDA) in 2001 for the treatment of chronic myeloid leukemia [38]. In the last years, a relevant number of preclinical studies and clinical trials have been proposed regarding the use of kinase inhibitors for HCC treatment (Table S1).

### 4.1. Multi-Kinase Inhibitors Currently Licensed for HCC

#### 4.1.1. Sorafenib (BAY 43-9006)

Sorafenib is a bi-aryl urea compound multikinase inhibitor that represses cancer cells growth and tumor angiogenesis and enhances the rate of apoptosis in many tumor models [39,40]. It works inhibiting the serine-threonine kinases c-Raf (Raf-1) and B-Raf; vascular endothelial growth factor receptor (VEGFR)-1, -2 and -3; platelet-derived growth factor receptors (PDGFR) α and β; the MEK; the cytokine receptor c-Kit; the fetal liver tyrosine kinases receptor 3 (FLT-3) and rearranged during transfection receptor tyrosine kinase (RET) [39,40,41]. The mechanism of inhibition is exerted thanks to an interaction between sorafenib and the ATP binding site of the targets [37,39].

Sorafenib has been evaluated in HCC because the cellular signaling mediated by the Raf-1 and VEGF pathways has been implicated in the molecular pathogenesis of HCC [42]. In 2007 sorafenib was approved for the treatment of advanced HCC patients with Child-Pugh A cirrhosis and good performance condition and BCLC stage C [43]. The approval was based on two phase III clinical trials [42,44]. In the first clinical trial (SHARP), patients with advanced HCC previously untreated with systemic therapy were administered with sorafenib at a dose of 400 mg twice a day or placebo. In patients treated with sorafenib median survival and time to radiological progression were approximately 90 days longer than those treated with placebo. The adverse effects were principally mild to moderate in severity (NCT00105443) [42]. In the second clinical study, patients with advanced HCC previously untreated with systemic therapy received oral sorafenib at a dose of 400 mg or placebo twice a day in 6-week cycles. Sorafenib demonstrated to be effective with tolerable adverse effects (NCT00492752) [44]. Several clinical trials are evaluating the use of sorafenib with other approaches (Table S1). Among these, two trials are currently investigating the use of sorafenib in combination with ICI. In particular, a phase II study (NCT03439891) is evaluating the combinational therapy composed by sorafenib and nivolumab (anti- programmed cell death protein 1 PD-1) in HCC patients not eligible for surgery. In addition, a phase I/II clinical study (NCT03211416) is evaluating the administration of sorafenib in combination with pembrolizumab (anti-PD-1) in metastatic HCC patients (Table S1).

#### 4.1.2. Regorafenib (BAY 73-4506)

Regorafenib is another urea-derived compound, in which the addition of a fluorine atom in the center phenyl ring makes its structure distinct from that of sorafenib [45]. This multikinase inhibitor showed anti-tumorigenic and anti-angiogenic behavior in pre-clinical and clinical studies [45,46]. Regorafenib strongly suppresses VEGFR-1, -2, and -3, PDGFRβ, TIE-2, fibroblast growth factor receptor 1 (FGFR1), the mutant oncogenic kinases KIT (CD117), RET, and B-Raf [45]. The mechanism of action of regorafenib is based on an interaction within the ATP binding site of the target molecules [47]. In April 2017, regorafenib was approved for the treatment of patients with advanced HCC whose disease progressed after sorafenib therapy [48]. This approval derived from a randomized, double-blind, placebo-controlled, phase III clinical trial (RESORCE) (NCT01774344) [49]. Several clinical trials are ongoing for the evaluation of regorafenib use including some trials investigating the combination with ICI such as pembrolizumab and avelumab (Table S1).

#### 4.1.3. Lenvatinib (E7080)

Lenvatinib is a multikinase urea derivative inhibitor [50]. In-vitro and in-vivo studies demonstrated that this molecule has potent anti-tumor and anti-angiogenesis activity [51,52]. This small molecule works inhibiting VEGFR-1, -2, and -3, FGFR1, 2, 3, and 4, PDGFRα, KIT, and RET [51,53]. A structural study demonstrated that lenvatinib inhibits its targets interacting with the ATP binding site [50]. On August 16, 2018, FDA approved lenvatinib for first-line therapy for unresectable HCC patients [54]. The approval originated from a randomized phase III study with 954 unresectable HCC patients evaluating lenvatinib versus sorafenib in terms of overall survival (NCT01761266) [55]. Several clinical trials are ongoing for the evaluation of lenvatinib use in HCC, some of them are in association with an immunotherapy approach based on nivolumab or pembrolizumab (Table S1).

#### 4.1.4. Cabozantinib (XL184, BMS-907351)

Cabozantinib is a strong tyrosine kinase inhibitor, ATP-competitor, able to block tyrosine-protein kinase Met (c-Met) and VEGFR-2 phosphorylation. It also acts by inhibiting tyrosine-protein kinase receptor UFO (AXL), c-Kit, RET and FLT-3 [56,57]. Cabozantinib is able to inhibit cellular migration, invasion and tumor cell proliferation in-vitro [56,57]. Tumor inhibition and metastasis prevention have also been shown in mouse models injected with HCC cell lines (MHCC97H, HepG2 and SK-HEP1) [56]. On 14 January 2019, FDA approved cabozantinib for progressive and metastatic medullary thyroid cancer, for advanced renal cell carcinoma (RCC), and for the treatment of HCC patients previously treated with sorafenib [58,59,60,61,62]. Several clinical trials are now investigating the use of cabozantinib for HCC treatment, some of them in combination with ICIs including nivolumab, durvalumab, atezolizumab, ipilimumab (Table S1).

### 4.2. Multi-Kinase Inhibitors not Currently Licensed for HCC

#### 4.2.1. Sunitinib (SU11248)

Sunitinib is an oral oxindol able to inhibit several tyrosine kinases namely VEGFR-1, and -2, PDGFRα/β, c-Kit, FLT-3, and RET [63,64]. Sunitinib works to prevent the phosphorylation of its targets [65]. Sunitinib displays anti-tumor and anti-angiogenetic activity in mouse xenograft models [64]. The use of sunitinib has been proposed for HCC treatment in 10 clinical trials (Table S1).

#### 4.2.2. Erlotinib (CP-358774, OSI-774)

Epidermal growth factor receptor (EGFR) autocrine pathway coordinates several activities relevant for tumor development and progression, including cell growth, angiogenesis, apoptosis, and metastasis [66]. In the HCC context, EGFR activity may repress the anti-tumor function of sorafenib, suggesting that the neutralization of EGFR may improve tumor response [67,68]. Erlotinib is a quinazoline derivative inhibitor of EGFR able to inhibit the kinase activity and also the auto-phosphorilation of EGFR [69]. This EGFR inhibitor demonstrated to have antitumor activity in-vitro and also in tumor xenograft mouse models [69,70]. There are 12 clinical trials proposed to investigate the use of erlotinib in combination with other approaches for HCC treatment (Table S1).

#### 4.2.3. Brivanib (BMS-540215)

Brivanib is a pyrrolatriazine-based compound inhibitor of VEGFR-2 and FGFR1 [71,72]. It competes with ATP for the binding in the ATP-binding domain [73]. Brivanib is able to suppress tumor growth in mouse models, including xenograft HCC mouse models, in keeping with the key role of VEGFR-2 and FGFR1 in the pathogenesis of HCC [42,73,74,75]. There are 8 clinical trials proposed to investigate the use of brivanib for HCC treatment (Table S1).

#### 4.2.4. Cediranib (AZD2171)

Cediranib is an indole-ether quinazoline compound, ATP-competitor and VEGFR-family inhibitor, with highest action against VEGFR-2, and it is also able to inhibit c-Kit and PDGFRβ [76]. It interferes in the endochondral ossification and ovary luteal phase in rats, events dependent on angiogenic pathways [76]. Cediranib inhibits human tumor xenograft growth in athymic mouse models both preventing the formation of novel vessels and causing the regression of vasculature [76]. Two clinical trials have been proposed to investigate the use of cediranib for HCC treatment (Table S1).

#### 4.2.5. Linifanib (ABT-869)

Linifanib is an ATP-competitive receptor tyrosine kinase inhibitor acting on the members of the VEGF and PDGF receptor families [77]. Linifanib is effective in several xenograft tumor models inhibiting their growth [77]. In particular, in HCC xenograft mouse models linifanib had a significant action in reducing tumor volume. Action that was enhanced when linifanib was administered in combination with rapamycin [78]. Two clinical trials have been proposed to investigate the use of linifanib to treat HCC.

#### 4.2.6. Nintedanib (BIBF1120)

Nintedanib is an indolinone and an angiokinase inhibitor able to interact with VEGFR-1, -2 and -3, PDGFR, FGFR and Src kinase family [79,80]. In xenograft tumor mouse models, nintedanib showed an inhibiting activity of VEGF-dependent cell proliferation [79]. In addition, nintedanib potentially regulates the Src homology region 2 domain-containing phosphatase-1 (SHP-1) autoinhibition causing an increase in signal transducer and activator of transcription 3 (STAT3) dephosphorylation through a kinase-independent mechanism, in xenograft HCC tumor mouse models [80]. Three clinical trials have been proposed to investigate the use of nintedanib for HCC treatment (Table S1).

#### 4.2.7. Refametinib (BAY 869766)

Refametinib is a MEK inhibitor able to bind MEK1/2 hydrophobic pocket, blocking the enzyme in the non-active form [81]. Regarding the HCC context, Schmieder et al. explored refametinib antitumor activity through different xenograft and allograft HCC tumor mouse models [82]. This study showed that refametinib is able to act as antiproliferative agent and to enhance survival in xenograft and allograft murine models [82]. Three clinical trials tested the efficacy of refametinib alone or in combination with sorafenib (Table S1).

#### 4.2.8. Vatalanib (PTK787/ZK222584)

Vatalanib has a potent inhibitory activity against VEGFR tyrosine kinases impairing their autophosphorylation activity and it is also able to inhibit PDGFRβ, c-Kit and colony stimulating factor 1 receptor (c-Fms) [83]. Vatalanib disrupts the neovasculature and inhibits capillary-like sprout formation in-vitro [83]. In the HCC context, vatalanib decreases microvessel density, inhibits the proliferation and induces apoptosis of tumor cells both in-vitro and in-vivo in HCC models [84,85,86]. Moreover, vatalanib plus interferon-α/5-fluorouracil (IFN/5-FU) decrease the expression of VEGFR-2 and reduce Akt/ERK/p38MAPK phosphorylation [86]. A phase I/II trial has been conducted to test vatalanib administered in combination with intravenous doxorubicin as therapy for patients with advanced HCC [87].

#### 4.2.9. Vandetanib (ZD6474)

Vandetanib is a 4-anilinoquinazoline with basic side chains at C-7 of the quinazoline nucleus able to inhibit VEGFR-2 and EGFR autophosphorylation [88,89]. Vandetanib has consistently anticancer activity in breast, colon, ovary, prostate and thyroid human tumor mouse models with reduction in neoangiogenesis in colon cancer xenograft models [88,89,90]. In HCC context, vandetanib, by inhibiting EGFR pathway, is able to block HCC cell line adhesion, proliferation, migration and invasiveness in-vitro [91]. In-vivo studies confirmed the antitumor activity of vandetanib in orthotopic and subcutaneous liver tumor nude mouse models [92]. Two clinical trials have been proposed to test the use of vandetanib as therapy for HCC (Table S1).

#### 4.2.10. Pazopanib (GW786034)

Pazopanib is an indazolypyrimidine and a multitargeted receptor tyrosine kinase inhibitor able to interact with VEGFR-1, -2 and -3, PDGFRα/β and c-Kit [93]. In particular, pazopanib inhibits VEGF-induced VEGFR-2 phosphorylation [93]. Pazopanib is able to inhibit the growth of different tumors in xenograft mice and presented antiangiogenic activity in-vivo [94,95]. In the HCC context, pazopanib inhibits HCC cell-derived tumors in xenograft mouse models, prolonging the survival of nude mice presenting tumors [96]. Two clinical trials investigated the use of pazopanib as therapy for different cancers, HCC among them (Table S1) [97].

#### 4.2.11. Tivantinib (ARQ 197)

Tivantinib is a highly-selective c-Met inhibitor that acts through a mechanism that is non-ATP-competitive, blocking the constitutive and ligand-mediated autophosphorylation of this receptor tyrosine kinase [98]. Tivantinib is able to induce apoptosis in cancer cell lines with c-Met constitutive activated and to inhibit tumor growth in xenograft cell-derived tumor mouse models [98]. The combination of tivantinib and sorafenib has been demonstrated to have an additive cytotoxic effect in HCC cell lines [99]. Subsequently, this receptor tyrosine kinase inhibitor can act on microtubule assembling [100,101] and glycogen synthase kinase 3 α and β [101,102]. There are 6 clinical trials that have been proposed to investigate the use of tivantinib for HCC treatment (Table S1).

#### 4.2.12. Apatinib (YN968D1)

Apatinib is a potent inhibitor of VEGFR-2, acting also against c-Kit and c-Src kinase activities and VEGFR-2, c-Kit and PDGFRβ phosphorylation [103]. Apatinib is able to inhibit proliferation, migration and tube formation of HUVEC cells in-vitro [103,104]. In addition, it is able to impair the growth of several human tumor xenograft mouse models (lung, colon and stomach) through its antiangiogenic effect, acting alone, or with a synergistic effect when administered in combination with chemotherapeutic agents [103]. In the HCC context, apatinib is able to inhibit HCC cell proliferation in-vitro in a dose-dependent manner and in relation with the level of VEGFR-2 expression [104]. Moreover, VEGF-dependent VEGFR-2-phospshorylation is inhibited by this drug. The antitumor action of apatinib has been correlated with a decrease in tumor microvessel densities and an increase in median survival in human xenograft mouse models [104]. Among the clinical trials investigating the use of apatinib in HCC (Table S1), 7 trials investigate the use of apatinib in combination with the anti-PD-1 antibody SHR-1210.

## 5. Antibodies and Peptibody Targeting Angiogenesis Factors

### 5.1. Bevacizumab (rhuMAB)

Bevacizumab is a humanized IgG1 monoclonal antibody (mAb) anti-VEGF that inhibits tumor growth [105,106]. It has been approved for the treatment of: recurrent, metastasized or refractory cervical cancer, metastasized colorectal cancer, recurrent or worsening glioblastoma, locally advanced, impossible to remove, metastasized or recurrent non-squamous non-small cell lung cancer, stage III or IV or recurrent ovarian epithelial, fallopian tube or primary peritoneal cancer, and metastasized renal cell carcinoma [107]. Twenty-two clinical trials have been proposed to investigate the use of bevacizumab for HCC treatment (Table S2). These clinical trials did not completely clarify the efficacy of bevacizumab in the context of HCC treatment (Table S2). Among these trials, several of them have been set up to evaluate the use of bevacizumab in combination with ICIs such as nivolumab, durvalumab or atezolizumab (Table S2).

### 5.2. Ramucirumab (IMC-1121B)

Ramucirumab is an anti-VEGFR-2 IgG1 mAb [108,109]. It is approved for the treatment of: advanced or metastatic stomach or gastroesophageal junction adenocarcinoma; metastatic colorectal cancer; metastatic non small cell lung carcinoma (NSCLC) [110]. Six clinical trials have been proposed to investigate the use of ramucirumab for HCC treatment (Table S2), including a trial in which the use of ramucirumab has been proposed in combination therapy with the ICI durvalumab (Table S2).

### 5.3. Trebananib (AMG386)

Trebananib is a recombinant protein made by the fusion of a Fc and a peptide (peptibody) able to interact with angiopoietin 1 or 2 (Ang-1/2), impairing their binding toTIE-2 angiopoietin receptor, thus inhibiting tumor angiogenesis [111,112,113]. In the first-in-human study, trebananib showed a tolerable safety profile in patients with advanced solid tumors [111]. Moreover, trebananib has been proposed in combination with sorafenib to treat advanced or inoperable HCC (Table S2).

## 6. Plant-Derived Products Targeting Angiogenesis

A large number of plant-derived compounds, such as polyphenols including resveratrol, curcumin and catechins have been reported to show an anti-angiogenic and anti-proliferative effect, and to suppress tumorigenesis in several tumors including HCC [114,115,116,117]. In particular, resveratrol affects tumor angiogenesis by inhibiting endothelial cell migration, proliferation and new blood vessel formation through the inhibition of fibroblast growth factor 2 (FGF2) and VEGF receptor-mediated activation of MAPK in endothelial cells [118]. Moreover, resveratrol inhibits VEGF expression and hypoxia-inducible factor-1 (HIF-1) alpha expression [119]. Curcumin displays anticancer and anti-inflammatory properties [120]. Moreover, curcumin inhibits endothelial cell proliferation induced by basic fibroblast growth factor (bFGF) and bFGF mediated neovascularization [121]. Of note, curcumin has been shown to reduce tumor neocapillary density and serum VEGF levels HCC mouse models [122]. Among flavonoids, genistein has been shown to inhibit bFGF mediated endothelial cell tube formation in-vitro [123]. Among alkaloids, castanospermine has been reported to inhibit migration and invasion of endothelial cells through the basement membrane, and to suppress tumor neovascularization and growth [124]. Colchicine has been shown to inhibit angiogensis in-vitro [125]. Vinblastine inhibits VEGF-mediated angiogenesis [1]. Sanguinarine has been shown to suppress VEGF-induced endotheilial cell migration and sprouting in-vitro and blood vessel formation in-vivo. Brucine and tylophorine has been shown to inhibit VEGF-mediated angiogenesis by suppressing downstream protein kinases, includimg ERK, PI3K, AKT, mTOR [126].

## 7. Conclusions

Antiangiogenic compounds are widely prescribed and continuously developed and investigated in clinical trials for solid tumor treatments although they generally offer a modest survival benefit of few weeks to months, with not frequent durable responses. Antiangiogenic therapy strategies are commonly used for the treatment of HCC. Sorafenib is the current option as standard first-line treatment in patients with advanced HCC as well as lenvatinib that has been recently approved as first-line treatment. Regorafenib is the most common option for patients who progress to sorafenib and cabozantinib is a recently approved alternative choice as a second-line treatment. Besides these compounds, there are other kinase inhibitors and antibodies with different mechanisms of action, whose use has been proposed in clinical trials for HCC treatment. However, it is not yet clear if the different mechanisms of action of these different compounds actually impact on clinical endpoints. In this context, to date, no advantage of one kinase inhibitor versus the other has been found in terms of overall survival in the only randomized trial facing the two kinase inhibitors sorafenib and regorafenib [49]. In the absence of other comparison data among different antiangiogenic molecules, information such as the toxicity profiles, characteristic of the study populations and the evaluation of biomarkers become crucial for the set-up of new clinical trials investigating the simultaneous or sequential combined use of these molecules. Moreover, antiangiogenic molecules can be efficiently included in treatment combinations with ICIs to ameliorate the efficacy of the chosen immunotherapeutic approach for the treatment of HCC patients [3,6,9,11,19,32,127].

A recently proposed point of view is represented by the need to define peculiar scheduling for the administration of antiangiogenic molecules to reach a transient pharmaceutical induced time interval in which tumor vessels show improved functionality rather than hypoxia, in a process called normalization of vasculature [3,7,35,128]. This restored functionality of tumor vessels is potentially useful to augment the capability of additional pharmaceutical compounds, such as ICIs, that can be employed in association with the antiangiogenic molecules to kill tumor cells [3,7]. In particular, preclinical studies have shown that there is an increased tumor infiltration by T cells in response to low doses of anti-VEGFR2 antibodies during the normalization window [15]. Moreover, it has been demonstrated that an antiangiogenic treatment, carried out several days before an immunotherapeutic treatment, is capable to increase the accumulation of T cells into the tumor, thus increasing anticancer efficacy with respect to immunotherapy alone [15,129]. Furthermore, loss of VEGF in T cells, resulting in vessel normalization, is capable to improve chemotherapeutic response in mice [130]. The extent of normalization varies among tumors, as well as with the duration of the treatment and the dosage of the drug, whereas, closure of the normalization window is marked by the development of adaptive tumor resistance and immunosuppression [35]. Alternative methods of vascularization, such as vessel cooption, and upregulation of alternative angiogenic pathways can also be used by the tumors to escape antiangiogenic therapies and to close the normalization window. For this reason, there is the need to critically evaluate the dose, duration and schedule of administration of antiangiogenic compounds for the use in treatment combinations with ICIs. In this context, results of clinical trials investigating the efficacy of treatment combinations of antiangiogenic molecules with ICIs, although in some cases encouraging, do not completely clarify the best dose and scheduling, and efficacy of these treatments.

Given the importance of the vascular normalization process, in particular in the context of treatment combinations of ICIs and antiangiogenic molecules, there is a raising need of biomarkers useful for the definition of when vascular normalization occurs [3,9,11,19]. Ever-increasing knowledge of the processes defining the link between angiogenesis and the immune system will hopefully allow the rapid introduction of therapeutic combination strategies in the routinely clinical practice for the treatment management of HCC patients.

## Figures and Tables

**Table 1:** Current phase in Hepatocellular carcinoma (HCC) of the different treatments/molecules described in the present review. Further details are reported in the Supplementary Materials (Tables S1 and S2).

Treatment/Molecule Name	Current Phase in HCC
TACE	Approved for clinical use
Sorafenib	Approved for clinical use
Regorafenib	Approved for clinical use
Lenvatinib	Approved for clinical use
Cabozantinib	Approved for clinical use
Sunitinib	Phase III
Erlotinib	Phase III
Brivanib	Phase III
Cediranib	Phase II
Linifanib	Phase III
Nintedanib	Phase II
Refametinib	Phase II
Vatalanib	Phase I/II
Vandetanib	Phase II
Pazopanib	Phase I
Tivantinib	Phase III
Apatinib	Phase III
Bevacizumab	Phase III
Ramucirumab	Phase III
Trebananib	Phase II

## Supplementary Materials

The following are available online at https://www.mdpi.com/2072-6694/11/8/1086/s1, Table S1: Clinical trials investigating the use of kinase inhibitors for hepatocellular carcinoma treatment described in the review, Table S2: Clinical trials investigating the use of antibodies and peptibody for hepatocellular carcinoma treatment described in the present review.
