# Supplementary material for: New Insight into Therapies Targeting Angiogenesis in Hepatocellular Carcinoma
Source: Cancers (Basel). 2019 Jul 31;11(8):1086. doi: 10.3390/cancers11081086 (PMC6721310; doi:10.3390/cancers11081086)
Supplement: Supplementary file 1 [file cancers-11-01086-s001.zip › suppl-final/cancers-552375-suppl-final.pdf]

# Supplementary Materials: New Insight into Therapies Targeting Angiogenesis in Hepatocellular Carcinoma

**Monica Mossenta, Davide Busato, Lorena Baboci, Federica Di Cintio, Giuseppe Toffoli and Michele Dal Bo**

## References

1. He, M.; Li, Q.; Zou, R.; Shen, J.; Fang, W.; Tan, G.; Zhou, Y.; Wu, X.; Xu, L.; Wei, W.; et al. Sorafenib Plus Hepatic Arterial Infusion of Oxaliplatin, Fluorouracil, and Leucovorin vs Sorafenib Alone for Hepatocellular Carcinoma With Portal Vein Invasion: A Randomized Clinical Trial. *JAMA Oncol.* **2019**, *5*, 953–960.
2. Bruix, J.; Qin, S.; Merle, P.; Granito, A.; Huang, Y.-H.; Bodoky, G.; Pracht, M.; Yokosuka, O.; Rosmorduc, O.; Breder, V.; et al. Regorafenib for patients with hepatocellular carcinoma who progressed on sorafenib treatment (RESORCE): a randomised, double-blind, placebo-controlled, phase 3 trial. *The Lancet* **2017**, *389*, 56–66.
3. Bruix, J.; Tak, W.-Y.; Gasbarrini, A.; Santoro, A.; Colombo, M.; Lim, H.-Y.; Mazzaferro, V.; Wiest, R.; Reig, M.; Wagner, A.; et al. Regorafenib as second-line therapy for intermediate or advanced hepatocellular carcinoma: Multicentre, open-label, phase II safety study. *Eur. J. Cancer* **2013**, *49*, 3412–3419.
4. Kudo, M.; Finn, R.S.; Qin, S.; Han, K.-H.; Ikeda, K.; Piscaglia, F.; Baron, A.; Park, J.-W.; Han, G.; Jassem, J.; et al. Lenvatinib versus sorafenib in first-line treatment of patients with unresectable hepatocellular carcinoma: a randomised phase 3 non-inferiority trial. *Lancet* **2018**, *391*, 1163–1173.
5. Ikeda, K.; Kudo, M.; Kawazoe, S.; Osaki, Y.; Ikeda, M.; Okusaka, T.; Tamai, T.; Suzuki, T.; Hisai, T.; Hayato, S.; et al. Phase 2 study of lenvatinib in patients with advanced hepatocellular carcinoma. *J. Gastroenterol.* **2017**, *52*, 512–519.
6. Abou-Alfa, G.K.; Meyer, T.; Cheng, A.-L.; El-Khoueiry, A.B.; Rimassa, L.; Ryoo, B.-Y.; Cicin, I.; Merle, P.; Chen, Y.; Park, J.-W.; et al. Cabozantinib in Patients with Advanced and Progressing Hepatocellular Carcinoma. *N. Engl. J. Med.* **2018**, *379*, 54–63.
7. Schöffski, P.; Gordon, M.; Smith, D.C.; Kurzrock, R.; Daud, A.; Vogelzang, N.J.; Lee, Y.; Scheffold, C.; Shapiro, G.I. Phase II randomised discontinuation trial of cabozantinib in patients with advanced solid tumours. *Eur. J. Cancer* **2017**, *86*, 296–304.
8. Kelley, R.K.; Verslype, C.; Cohn, A.L.; Yang, T.-S.; Su, W.-C.; Burris, H.; Braiteh, F.; Vogelzang, N.; Spira, A.; Foster, P.; et al. Cabozantinib in hepatocellular carcinoma: results of a phase 2 placebo-controlled randomized discontinuation study. *Ann. Oncol.* **2017**, *28*, 528–534.
9. Sahani, D.V.; Jiang, T.; Hayano, K.; Duda, D.G.; Catalano, O.A.; Ancukiewicz, M.; Jain, R.K.; Zhu, A.X. Magnetic resonance imaging biomarkers in hepatocellular carcinoma: association with response and circulating biomarkers after sunitinib therapy. *J. Hematol. Oncol.J Hematol Oncol* **2013**, *6*, 51.
10. Faivre, S.; Raymond, E.; Boucher, E.; Douillard, J.; Lim, H.Y.; Kim, J.S.; Zappa, M.; Lanzalone, S.; Lin, X.; DePrimo, S.; et al. Safety and efficacy of sunitinib in patients with advanced hepatocellular carcinoma: an open-label, multicentre, phase II study. *Lancet Oncol.* **2009**, *10*, 794–800.
11. Cheng, A.-L.; Kang, Y.-K.; Lin, D.-Y.; Park, J.-W.; Kudo, M.; Qin, S.; Chung, H.-C.; Song, X.; Xu, J.; Poggi, G.; et al. Sunitinib Versus Sorafenib in Advanced Hepatocellular Cancer: Results of a Randomized Phase III Trial. *J. Clin. Oncol.* **2013**, *31*, 4067–4075.
12. Chiorean, E.G.; Ramasubbaiah, R.; Yu, M.; Picus, J.; Bufill, J.A.; Tong, Y.; Coleman, N.; Johnston, E.L.; Currie, C.; Loehrer, P.J. Phase II trial of erlotinib and docetaxel in advanced and refractory hepatocellular and biliary cancers: Hoosier Oncology Group GI06-101. *The Oncologist* **2012**, *17*, 13.
13. Zhu, A.X.; Rosmorduc, O.; Evans, T.R.J.; Ross, P.J.; Santoro, A.; Carrilho, F.J.; Bruix, J.; Qin, S.; Thuluvath, P.J.; Llovet, J.M.; et al. SEARCH: a phase III, randomized, double-blind, placebo-controlled trial of sorafenib plus erlotinib in patients with advanced hepatocellular carcinoma. *J. Clin. Oncol.* **2015**, *33*, 559–566.
14. Johnson, P.J.; Qin, S.; Park, J.-W.; Poon, R.T.P.; Raoul, J.-L.; Philip, P.A.; Hsu, C.-H.; Hu, T.-H.; Heo, J.; Xu, J.; et al. Brivanib Versus Sorafenib As First-Line Therapy in Patients With Unresectable, Advanced Hepatocellular Carcinoma: Results From the Randomized Phase III BRISK-FL Study. *J. Clin. Oncol.* **2013**, *31*, 3517–3524.
15. Park, J.-W.; Finn, R.S.; Kim, J.S.; Karwal, M.; Li, R.K.; Ismail, F.; Thomas, M.; Harris, R.; Baudelet, C.; Walters, I.; et al. Phase II, Open-Label Study of Brivanib as First-Line Therapy in Patients with Advanced Hepatocellular Carcinoma. *Clin. Cancer Res.* **2011**, *17*, 1973–1983.

16. Kudo, M.; Han, G.; Finn, R.S.; Poon, R.T.P.; Blanc, J.-F.; Yan, L.; Yang, J.; Lu, L.; Tak, W.-Y.; Yu, X.; et al. Brivanib as adjuvant therapy to transarterial chemoembolization in patients with hepatocellular carcinoma: A randomized phase III trial. *Hepatology* **2014**, *60*, 1697–1707.
17. El-Khoueiry, A.; Posey, J.A.; Castillo Ferrando, J.R.; Krishnamurthi, S.S.; Syed, S.; Kollia, G.; Walters, I.; Fischer, B.S.; Masson, E. The effects of liver impairment on the pharmacokinetics of brivanib, a dual inhibitor of fibroblast growth factor receptor and vascular endothelial growth factor receptor tyrosine kinases. *Cancer Chemother. Pharmacol.* **2013**, *72*, 53–64.
18. Llovet, J.M.; Decaens, T.; Raoul, J.-L.; Boucher, E.; Kudo, M.; Chang, C.; Kang, Y.-K.; Assenat, E.; Lim, H.-Y.; Boige, V.; et al. Brivanib in Patients With Advanced Hepatocellular Carcinoma Who Were Intolerant to Sorafenib or for Whom Sorafenib Failed: Results From the Randomized Phase III BRISK-PS Study. *J. Clin. Oncol.* **2013**, *31*, 3509–3516.
19. Zhu, A.X.; Ancukiewicz, M.; Supko, J.G.; Sahani, D.V.; Blaszkowsky, L.S.; Meyerhardt, J.A.; Abrams, T.A.; McCleary, N.J.; Bhargava, P.; Muzikansky, A.; et al. Efficacy, safety, pharmacokinetics, and biomarkers of cediranib monotherapy in advanced hepatocellular carcinoma: a phase II study. *Clin. Cancer Res.* **2013**, *19*, 1557–1566.
20. Toh, H.C.; Chen, P.-J.; Carr, B.I.; Knox, J.J.; Gill, S.; Ansell, P.; McKeegan, E.M.; Dowell, B.; Pedersen, M.; Qin, Q.; et al. Phase 2 trial of linifanib (ABT-869) in patients with unresectable or metastatic hepatocellular carcinoma. *Cancer* **2013**, *119*, 380–387.
21. Cainap, C.; Qin, S.; Huang, W.-T.; Chung, I.J.; Pan, H.; Cheng, Y.; Kudo, M.; Kang, Y.-K.; Chen, P.-J.; Toh, H.-C.; et al. Linifanib versus Sorafenib in patients with advanced hepatocellular carcinoma: results of a randomized phase III trial. *J. Clin. Oncol.* **2015**, *33*, 172–179.
22. Yen, C.-J.; Kim, T.-Y.; Feng, Y.-H.; Chao, Y.; Lin, D.-Y.; Ryoo, B.-Y.; Huang, D.C.-L.; Schnell, D.; Hocke, J.; Loembé, A.-B.; et al. A Phase I/Randomized Phase II Study to Evaluate the Safety, Pharmacokinetics, and Efficacy of Nintedanib versus Sorafenib in Asian Patients with Advanced Hepatocellular Carcinoma. *Liver Cancer* **2018**, *7*, 165–178.
23. Okusaka, T.; Otsuka, T.; Ueno, H.; Mitsunaga, S.; Sugimoto, R.; Muro, K.; Saito, I.; Tadayasu, Y.; Inoue, K.; Loembé, A.-B.; et al. Phase I study of nintedanib in Japanese patients with advanced hepatocellular carcinoma and liver impairment. *Cancer Sci.* **2016**, *107*, 1791–1799.
24. Palmer, D.H.; Ma, Y.T.; Peck-Radosavljevic, M.; Ross, P.; Graham, J.; Fartoux, L.; Deptala, A.; Studeny, M.; Schnell, D.; Hocke, J.; et al. A multicentre, open-label, phase-I/randomised phase-II study to evaluate safety, pharmacokinetics, and efficacy of nintedanib vs. sorafenib in European patients with advanced hepatocellular carcinoma. *Br. J. Cancer* **2018**, *118*, 1162–1168.
25. Lim, H.Y.; Merle, P.; Weiss, K.H.; Yau, T.; Ross, P.; Mazzaferro, V.; Blanc, J.-F.; Ma, Y.T.; Yen, C.J.; Kocsis, J.; et al. Phase II Studies with Refametinib or Refametinib plus Sorafenib in Patients with RAS-Mutated Hepatocellular Carcinoma. *Clin. Cancer Res.* **2018**, *24*, 4650–4661.
26. Lim, H.Y.; Heo, J.; Choi, H.J.; Lin, C.-Y.; Yoon, J.-H.; Hsu, C.; Rau, K.-M.; Poon, R.T.P.; Yeo, W.; Park, J.-W.; et al. A phase II study of the efficacy and safety of the combination therapy of the MEK inhibitor refametinib (BAY 86-9766) plus sorafenib for Asian patients with unresectable hepatocellular carcinoma. *Clin. Cancer Res.* **2014**, *20*, 5976–5985.
27. Yau, T.; Chen, P.-J.; Chan, P.; Curtis, C.M.; Murphy, P.S.; Suttle, A.B.; Gauvin, J.; Hodge, J.P.; Dar, M.M.; Poon, R.T. Phase I dose-finding study of pazopanib in hepatocellular carcinoma: evaluation of early efficacy, pharmacokinetics, and pharmacodynamics. *Clin. Cancer Res.* **2011**, *17*, 6914–6923.
28. Rimassa, L.; Assenat, E.; Peck-Radosavljevic, M.; Pracht, M.; Zagonel, V.; Mathurin, P.; Rota Caremoli, E.; Porta, C.; Daniele, B.; Bolondi, L.; et al. Tivantinib for second-line treatment of MET-high, advanced hepatocellular carcinoma (METIV-HCC): a final analysis of a phase 3, randomised, placebo-controlled study. *Lancet Oncol.* **2018**, *19*, 682–693.
29. Okusaka, T.; Aramaki, T.; Inaba, Y.; Nakamura, S.; Morimoto, M.; Moriguchi, M.; Sato, T.; Ikawa, Y.; Ikeda, M.; Furuse, J. Phase I study of tivantinib in Japanese patients with advanced hepatocellular carcinoma: Distinctive pharmacokinetic profiles from other solid tumors. *Cancer Sci.* **2015**, *106*, 611–617.
30. Santoro, A.; Rimassa, L.; Borbath, I.; Daniele, B.; Salvagni, S.; Van Laethem, J.L.; Van Vlierberghe, H.; Trojan, J.; Kolligs, F.T.; Weiss, A.; et al. Tivantinib for second-line treatment of advanced hepatocellular carcinoma: a randomised, placebo-controlled phase 2 study. *Lancet Oncol.* **2013**, *14*, 55–63.
31. Santoro, A.; Simonelli, M.; Rodriguez-Lope, C.; Zucali, P.; Camacho, L.H.; Granito, A.; Senzer, N.; Rimassa, L.; Abbadessa, G.; Schwartz, B.; et al. A Phase-1b study of tivantinib (ARQ 197) in adult patients with hepatocellular carcinoma and cirrhosis. *Br. J. Cancer* **2013**, *108*, 21–24.
32. Puzanov, I.; Sosman, J.; Santoro, A.; Saif, M.W.; Goff, L.; Dy, G.K.; Zucali, P.; Means-Powell, J.A.; Ma, W.W.; Simonelli, M.; et al. Phase 1 trial of tivantinib in combination with sorafenib in adult patients with advanced solid tumors. *Invest. New Drugs* **2015**, *33*, 159–168.

33. Xu, J.; Zhang, Y.; Jia, R.; Yue, C.; Chang, L.; Liu, R.; Zhang, G.; Zhao, C.; Zhang, Y.; Chen, C.; et al. Anti-PD-1 Antibody SHR-1210 Combined with Apatinib for Advanced Hepatocellular Carcinoma, Gastric, or Esophagogastric Junction Cancer: An Open-label, Dose Escalation and Expansion Study. *Clin. Cancer Res.* **2019**, *25*, 515–523.
34. Govindarajan, R.; Siegel, E.; Makhoul, I.; Williamson, S. Bevacizumab and Erlotinib in Previously Untreated Inoperable and Metastatic Hepatocellular Carcinoma: *Am. J. Clin. Oncol.* **2013**, *36*, 254–257.
35. Kaseb, A.O.; Morris, J.S.; Iwasaki, M.; Al-Shamsi, H.O.; Raghav, K.P.S.; Girard, L.; Cheung, S.; Nguyen, V.; Elsayes, K.M.; Xiao, L.; et al. Phase II trial of bevacizumab and erlotinib as a second-line therapy for advanced hepatocellular carcinoma. *OncoTargets Ther.* **2016**, *9*, 773–780.
36. Philip, P.A.; Mahoney, M.R.; Holen, K.D.; Northfelt, D.W.; Pitot, H.C.; Picus, J.; Flynn, P.J.; Erlichman, C. Phase 2 Study of Bevacizumab Plus Erlotinib in Patients With Advanced Hepatocellular Cancer. *Cancer* **2012**, *118*, 2424–2430.
37. Knox, J.J.; Qin, R.; Strosberg, J.R.; Tan, B.; Kaubisch, A.; El-Khoueiry, A.B.; Bekaii-Saab, T.S.; Rousey, S.R.; Chen, H.X.; Erlichman, C. A phase II trial of bevacizumab plus temsirolimus in patients with advanced hepatocellular carcinoma. *Invest. New Drugs* **2015**, *33*, 241–246.
38. Britten, C.D.; Gomes, A.S.; Wainberg, Z.A.; Elashoff, D.; Amado, R.; Xin, Y.; Busuttil, R.W.; Slamon, D.J.; Finn, R.S. Transarterial chemoembolization plus or minus intravenous bevacizumab in the treatment of hepatocellular cancer: A pilot study. *BMC Cancer* **2012**, *12*, 16.
39. Choo, S.P.; Chowbay, B.; Ng, Q.S.; Thng, C.H.; Lim, C.; Hartono, S.; Koh, T.S.; Huynh, H.; Poon, D.; Ang, M.K.; et al. A Phase 1 dose-finding and pharmacodynamic study of rapamycin in combination with bevacizumab in patients with unresectable hepatocellular carcinoma. *Eur. J. Cancer* **2013**, *49*, 999–1008.
40. Hubbard, J.M.; Mahoney, M.R.; Loui, W.S.; Roberts, L.R.; Smyrk, T.C.; Gatalica, Z.; Borad, M.; Kumar, S.; Alberts, S.R. Phase I/II Randomized Trial of Sorafenib and Bevacizumab as First-Line Therapy in Patients with Locally Advanced or Metastatic Hepatocellular Carcinoma: North Central Cancer Treatment Group trial N0745 (Alliance). *Target. Oncol.* **2017**, *12*, 201–209.
41. Kemeny, N.E.; Schwartz, L.; Gönen, M.; Yopp, A.; Gultekin, D.; D'Angelica, M.I.; Fong, Y.; Haviland, D.; Gewirtz, A.N.; Allen, P.; et al. Treating Primary Liver Cancer with Hepatic Arterial Infusion of Floxuridine and Dexamethasone: Does the Addition of Systemic Bevacizumab Improve Results? *Oncology* **2011**, *80*, 153–159.
42. Zhu, A.X.; Kang, Y.-K.; Yen, C.-J.; Finn, R.S.; Galle, P.R.; Llovet, J.M.; Assenat, E.; Brandi, G.; Lim, H.Y.; Pracht, M.; et al. REACH-2: A randomized, double-blind, placebo-controlled phase 3 study of ramucirumab versus placebo as second-line treatment in patients with advanced hepatocellular carcinoma (HCC) and elevated baseline alpha-fetoprotein (AFP) following first-line sorafenib. *J. Clin. Oncol.* **2018**, *36*, 4003–4003.
43. Zhu, A.X.; Park, J.O.; Ryoo, B.-Y.; Yen, C.-J.; Poon, R.; Pastorelli, D.; Blanc, J.-F.; Chung, H.C.; Baron, A.D.; Pfiffer, T.E.F.; et al. Ramucirumab versus placebo as second-line treatment in patients with advanced hepatocellular carcinoma following first-line therapy with sorafenib (REACH): a randomised, double-blind, multicentre, phase 3 trial. *Lancet Oncol.* **2015**, *16*, 859–870.
44. Zhu, A.X.; Baron, A.D.; Malfertheiner, P.; Kudo, M.; Kawazoe, S.; Pezet, D.; Weissinger, F.; Brandi, G.; Barone, C.A.; Okusaka, T.; et al. Ramucirumab as Second-Line Treatment in Patients With Advanced Hepatocellular Carcinoma: Analysis of REACH Trial Results by Child-Pugh Score. *JAMA Oncol.* **2017**, *3*, 235–243.
45. Chau, I.; Peck-Radosavljevic, M.; Borg, C.; Malfertheiner, P.; Seitz, J.F.; Park, J.O.; Ryoo, B.-Y.; Yen, C.-J.; Kudo, M.; Poon, R.; et al. Ramucirumab as second-line treatment in patients with advanced hepatocellular carcinoma following first-line therapy with sorafenib: Patient-focused outcome results from the randomised phase III REACH study. *Eur. J. Cancer* **2017**, *81*, 17–25.
46. Kudo, M.; Hatano, E.; Ohkawa, S.; Fujii, H.; Masumoto, A.; Furuse, J.; Wada, Y.; Ishii, H.; Obi, S.; Kaneko, S.; et al. Ramucirumab as second-line treatment in patients with advanced hepatocellular carcinoma: Japanese subgroup analysis of the REACH trial. *J. Gastroenterol.* **2017**, *52*, 494–503.
47. Harding, J.J.; Bendell, J.C.; Fuchs, C.S.; Wang, X.; Wacheck, V.; Zhu, A.X. Emibetuzumab plus ramucirumab: Simultaneous targeting of MET and VEGFR-2 in patients with advanced hepatocellular cancer in a phase 1b/2 study. *J. Clin. Oncol.* **2016**, *34*, 300–300.
48. Abou-Alfa, G.K.; Blanc, J.-F.; Miles, S.; Ganten, T.; Trojan, J.; Cebon, J.; Liem, A.K.; Lipton, L.; Gupta, C.; Wu, B.; et al. Phase II Study of First-Line Trebananib Plus Sorafenib in Patients with Advanced Hepatocellular Carcinoma. *The Oncologist* **2017**, *22*, 780-e65.


© 2019 by the authors. Licensee MDPI, Basel, Switzerland. This article is an open access article distributed under the terms and conditions of the Creative Commons Attribution (CC BY) license (http://creativecommons.org/licenses/by/4.0/).
